# Pharmacological effects of *N*-[2-[[2-[2-[(2,6-dichlorophenyl)amino]phenyl]acetyl]oxy]ethyl]hyaluronamide (diclofenac Etalhyaluronate, SI-613), a novel sodium hyaluronate derivative chemically linked with diclofenac

**DOI:** 10.1186/s12891-018-2077-8

**Published:** 2018-05-22

**Authors:** Keiji Yoshioka, Tomochika Kisukeda, Ryoji Zuinen, Yosuke Yasuda, Kenji Miyamoto

**Affiliations:** Central Research Lab., Research & Development Div., Seikagaku Corporation, 1253, Tateno 3-chome, Higashiyamato-shi, Tokyo 207-0021 Japan

**Keywords:** Osteoarthritis, Hyaluronan, Diclofenac etalhyaluronate, Sustained-release, Conjugate technology

## Abstract

**Background:**

Osteoarthritis (OA) is the most common joint disorder worldwide and one of the leading causes of disability in the elderly. We have investigated the novel sodium hyaluronate derivative chemically linked with diclofenac (DF), diclofenac etalhyaluronate (SI-613), which is a potentially safer and more effective treatment for OA knee pain. In this study, we evaluated the pharmacological effects of SI-613 in experimental arthritis models.

**Methods:**

We compared the analgesic and anti-inflammatory effects of intra-articularly administered SI-613, hyaluronic acid (HA), and of orally administered diclofenac sodium (DF-Na) in rat silver nitrate-induced arthritis model and rabbit antigen-induced arthritis model.

**Results:**

A single intra-articular (IA) administration of SI-613 significantly suppressed pain responses in rats in a dose-dependent manner. The analgesic effects were greater than those of HA, a mixture of DF-Na and HA, or an oral once-daily administration of DF-Na. In the rabbit arthritis model, SI-613 significantly reduced knee joint swelling compared with that in the control group on day 1 after a single IA injection. This significant anti-inflammatory effect was observed until day 28. In the pharmacokinetic study, the DF concentration in the synovium after SI-613 administration reached its maximum concentration of 311.6 ng/g on day 1, and gradually declined to 10 ng/g by day 28. It fell below the lower limit of quantification on day 35. Thus, a clear correlation was found between pharmacokinetics and pharmacodynamics. These results demonstrate that SI-613 exerts its long-lasting and potent anti-inflammatory effect by sustainable release of DF in the knee joint tissues.

**Conclusion:**

A single IA injection of SI-613 was shown to exert analgesic and anti-inflammatory effects for 28 days in non-clinical pharmacological studies, suggesting that SI-613 will be a promising candidate in the treatment of osteoarthritis pain.

## Background

Osteoarthritis (OA) is the most common joint disorder worldwide and one of the leading causes of disability in the elderly [1]. Treatment for knee OA aims to relieve pain and improve function, in order to mitigate reductions in physical activity. The mainstay of pharmacological therapy for OA includes acetaminophen, nonsteroidal anti-inflammatory drugs (NSAIDs) (oral and topical), cyclooxygenase-2 (COX-2) inhibitors, and IA therapies such as intra-articular sodium hyaluronate (IA-HA) injections and intra-articular-steroid (IA-steroid) injections.

Oral NSAIDs are widely prescribed for the treatment of OA pain. Nevertheless, upper gastrointestinal tract complications have been reported in patients who received long-term oral NSAIDs [2]. NSAIDs are also considered to have limited efficacy for OA pain relief. In the 1990s, a number of selective COX-2 inhibitors were developed to reduce the adverse events of NSAIDs. However, most of them were withdrawn from the market after the cardiovascular adverse effects of COX-2 inhibitors were reported in 2004 [3]. Meanwhile, oral NSAIDs including diclofenac sodium (DF-Na) were also reported to have the same concerns as COX-2 inhibitors [4, 5]. Thereafter, a few topical NSAIDs formulations were developed and launched for the relief of OA pain. Topical NSAIDs formulations, such as diclofenac sodium 1% gel, have equivalent efficacy and fewer adverse events compared with oral NSAIDs [6–8]. The intra-articular (IA) injection of hyaluronic acid (HA) is a recognized treatment for pain associated with symptomatic knee OA [9–11]. The pain relief afforded by IA-HA injections is long lasting and often lasts longer than 13 weeks [12, 13]. However, the efficacy of IA-HA injections is moderate compared to that of IA-steroids or oral NSAIDs. Therefore, the profiles of the next generation of OA therapeutics should be potent and longer lasting with higher safety, which will improve the quality of life for OA patients.

**Fig. 1:**
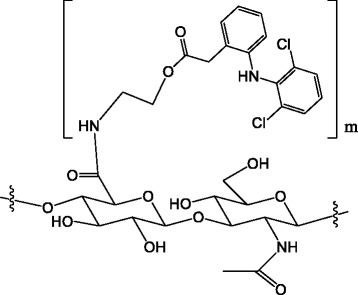
Chemical structure of *N*-[2-[[2-[2-[(2,6-dichlorophenyl)amino]phenyl]acetyl]oxy]ethyl]hyaluronamide (diclofenac etalhyaluronate, SI-613)

In the pursuit of the next generation of OA therapeutics, we developed a novel conjugated compound, SI-613. It is a novel derivative of high-molecular-weight fermented HA (600,000 to 1,200,000 Da), which tethers the NSAID diclofenac (DF) via a 2-aminoethanol linker extended from glucuronic acid moieties (Fig. 1). SI-613 gradually releases DF by hydrolytic cleavage of the ester linkage in a pH-dependent manner. Furthermore, SI-613 releases DF locally in a sustained manner and remains in the joint for a long period, similar to the existing IA-HA injection. It is expected to be more advantageous compared to IA-HA injections and NSAIDs in terms of efficacy and duration.

In the present study, we investigated the pharmacological effects of the IA administration of SI-613 and compared them with those of p.o. DF-Na and IA-HA administration. Furthermore, we investigated the pharmacokinetics of intra-articularly administered SI-613.

## Methods

### Animals

Male Sprague-Dawley (SD) rats (5 weeks old) were obtained from Charles River Laboratories Japan Inc. (Tokyo, Japan). Male New Zealand White rabbits (12–15 weeks old) were obtained from Oriental Yeast Co., Ltd. (Tokyo, Japan). Animals were maintained under specific pathogen free conditions at a room temperature of 23 ± 3 °C and air humidity of 50 ± 20% on a 12-h/12-h light/dark cycle. Animals were quarantined and acclimatized to the environmental conditions for 1 week.

### Rat model of silver nitrate-induced arthritic pain

The silver nitrate-induced arthritic pain model is known as a subacute arthritis model, in which the inflammatory response and pain last for at least 3 days following injury. This model involves the activation of prostaglandin pathways and has been used for evaluating the analgesic effects of various NSAIDs or kappa-opioid receptor agonist [14–18]. Overall, 136 male Sprague-Dawley (SD) rats were obtained from Charles River Laboratories Japan Inc. General anesthesia was maintained by the inhalation of isoflurane (Forane; Dainippon Sumitomo Pharma Co., Ltd., Osaka, Japan). Silver nitrate solution (1%, Wako Pure Chemical Industries, Ltd., Osaka, Japan) at a volume of 50 μL/joint was injected in the knee joint cavity of the left hindlimbs of rats. Animals with no abnormalities were allocated to four groups in each study based on the weight-bearing rate of the inflamed joints and pain score at the time of allocation, by using stratified continuous randomization. Test substances were administered on the day following the arthritis induction. In the first study, SI-613 (contents of DF; 11.8% (*w*/w), manufactured by Seikagaku Corporation, Tokyo, Japan) at doses of 0.05, 0.15, and 0.5 mg/50 μL/joint (5.9, 17.7, and 59 μg/joint in DF equivalent) or phosphate-buffered saline (PBS) was once injected into the joint cavities of the left hindlimbs for the dose-response assessments.

In the second study, SI-613 (0.5 mg/joint, 59 μg/joint in DF equivalent), HA (0.5 mg/joint, Seikagaku Corporation), a mixture of DF-Na (59 μg/joint, Wako Pure Chemical Industries, Ltd.) and HA (0.5 mg/joint) (DF-Na + HA), or PBS was administered in the same manner for the proof of concept. The doses of DF-Na and HA were set at those of respective components in SI-613 formulation. The DF-Na solution (1 mg/mL) was prepared using the Water for Injection (Otsuka Pharmaceutical Factory, Inc., Tokyo, Japan) and administered orally at a dose of 2 mg/kg (approximately 0.3 mg/body) once daily for 3 days. The oral DF-Na dose, 2 mg/kg, was set in accordance with the dose for adult humans with a body weight of 50 kg, on the assumption that the maximum daily dose in clinical practice administered to patients with OA or rheumatoid arthritis would be 100 mg. It has been reported that DF-Na exerted anti-inflammatory effects in rats when administered at this dose [19].

Pain was assessed under blinded conditions by scoring pain-related behaviors based on the following criteria and measuring the weight-bearing rates of hindlimbs with a load-measuring device (Tokken Inc., Chiba, Japan) at a same time each day for 3 days after the injection of the test materials.

The criteria for assigning pain scores were as follows: 0; normal, 1; mild claudication with lifting the foot, 2; severe claudication with completely closing the toe, 3; walking on three legs. The weight-bearing rate was calculated using the following formula:

Weight-bearing rate (%) = Mean weight-bearing load on inflamed leg (g) / Body weight (g) × 100.

In addition, the prostaglandin E_2_ (PGE_2_) content, which plays a critical role in inducing inflammation and pain associated with arthritis, was determined in the synovial fluid (SF). Briefly, SI-613 (0.5 mg/joint) or PBS was administered into the joint cavity of the left hindlimb. After assessing the severity of pain, animals were sacrificed by exsanguination under 2% isoflurane anesthesia 1, 2 and 3 days after administration, respectively. The SF was collected by washing the joint cavity with saline (Otsuka Pharmaceutical Factory, Inc.) containing indomethacin (Indacin, MSD K. K., Tokyo, Japan), which was effective to prevent joint puncturing-induced production of PGE_2_ (unpublished result). The PGE_2_ content in the SF was measured with a High Sensitivity PGE_2_ Correlate-EIA kit (Assay Designs Inc., Ann Arbor, MI).

### Effect of SI-613 on the PGE_2_ content in the SF of rabbits with antigen-induced arthritis

The anti-inflammatory effects of SI-613 were evaluated in a rabbit arthritis model induced by ovalbumin (OVA) [20–22], and compared with those of orally-administered DF-Na or the active chemical compositions of SI-613. To prepare the anesthetic, saline (2 mL), midazolam (1 mL, 5 mg/mL, Astellas Pharma Inc., Tokyo, Japan), xylazine (2 mL, 0.02 g/mL, Bayer Medical Ltd., Tokyo, Japan), and butorphanol tartrate (1 mL, 5 mg/mL, Meiji Seika Kaisha, Ltd., Tokyo, Japan) were mixed. The anesthetic was administered intravenously to each animal at a volume of 1 mL/body. OVA (Sigma-Aldrich Co., St*.* Louis, MO) emulsion with Freund’s complete adjuvant (FCA; CAPPEL Laboratories Inc., Cochranville, PA) was injected intradermally into the backs of 80 male rabbits at a dose of 5 mg/animal twice at an interval of 13 or 14 days. Twenty-three days after the first immunization, 1% OVA solution was injected in the joint cavities of the left hindlimb at a volume of 500 μL/joint to induce arthritis. Two days after the induction of arthritis, test materials of 5 mg/joint SI-613, a mixture of 0.59 mg/joint DF-Na and 5 mg/joint HA (DF-Na + HA), or PBS (control) were administered at a volume of 500 μL/joint in the knee joint cavity. DF-Na was orally administered at a dose of 2 mg/kg. Animals were sacrificed by exsanguination under 2% isoflurane anesthesia 3 and 72 h after administration, respectively. The SF was collected by washing the joint cavity twice with saline (Otsuka Pharmaceutical Factory, Inc.) containing 20 μg/mL of indomethacin (Indacin, MSD K. K.) at 3 or 72 h after the administration of the test materials. The PGE_2_ content in the SF was determined as a mechanism biomarker for the anti-inflammatory effect using a High Sensitivity Prostaglandin E_2_ Enzyme Immunoassay Kit (Assay Designs Inc.).

### Long-lasting anti-inflammatory effect of SI-613 on antigen-induced arthritis in rabbits

The duration and efficacy of the anti-inflammatory effect of SI-613 was studied in OVA-induced arthritis rabbits [20–22]. Two days after the arthritis induction, the knee joint diameter in all 60 rabbits was measured with a digital thickness gauge (Teclock Corp., Nagano, Japan). The joint swelling was expressed as the difference in millimeters between the inflamed (left) and non-inflamed (right) knee joint diameters. The same researcher evaluated the knee joint swelling on all groups on all days. Fifteen animals (knee joint swelling was not more than 7.30 mm) and 5 animals (knee joint swelling was not less than 10.40 mm) were excluded. Forty animals were divided into two groups of 20 animals each by the stratified random sampling method based on the knee joint swelling and body weight of the day, and were administered with 5 mg/joint SI-613 or PBS at a volume of 500 μL/joint into the joint cavities. The knee joint swelling was evaluated the day before (day 0), and on days 1, 3, 7, 14, 21, 28, 35, and 42 after a single injection of the test materials. The knee joint swelling was expressed as the difference in the width of the right and left knee joint.

### Distribution of DF in knee tissues after a single IA administration of SI-613 and its chemical compositions in rabbits: Short-term study

Arthritis was induced in the 12 rabbits as described above. Two days after the induction of arthritis, 5 mg/joint SI-613, a mixture of 0.59 mg/joint DF-Na and 5 mg/joint HA (DF-Na + HA), or PBS was administered to the knee joint cavity at a volume of 500 μL/joint. DF-Na was orally administered at a dose of 2 mg/kg. The concentrations of free DF in the synovium and synovial lavage fluid were measured by high performance liquid chromatography coupled with tandem mass spectrometry (LC-MS/MS) at 3 and 72 h after a single IA administration and after a single oral administration of the test materials, respectively. Moreover, plasma concentrations of DF were determined to compare the systemic exposure of DF after the administration of SI-613 with that of the other compounds. The synovium was homogenized in 40-fold volume (40 mL for 1 g tissue) of 10 mM ammonium formate (pH 6.0)/methanol (3:2, *v*/v) on an ice bath. Then, free DF in the homogenate was extracted with *tert*-butyl methyl ether-1% acetic acid (6:1, *v*/v). Free DF in the synovial fluid or plasma was adsorbed to an Oasis HLB cartridge (30 mg/1 cm^3^, Waters Corporation, Milford, MA) and eluted with methanol. The internal standard of deuterium-labeled diclofenac (diclofenac-d7) was added to each sample. The extract was loaded onto a CAPCELL PAK C18 MG HPLC column (Shiseido Co. Ltd., Tokyo, Japan, column size: 4.6 mm × 35 mm, particle size: 5 μm) at 40 °C, and eluted with 10 mM ammonium formate (pH 6.0)/methanol (2:3, *v*/v) at a flow rate of 0.5 mL/minute. For mass detection, we used a QTRAP^®^ 5500 System (AB SCIEX, Framingham, MA) equipped with an electrospray ionization (ESI) source in positive ions in multiple reaction monitoring (MRM) mode. Linear calibration (*r* > 0.999) was attained at 5–1000 ng/g for the synovium and at 1–200 ng/mL for the synovial fluid and plasma. The extraction efficiencies of diclofenac were 84.2–92.9% for the synovium, 95.2–100.3% for the synovial fluid, and 94.2–96.5% for the plasma.

### Evaluation of DF in knee tissues after a single IA administration of SI-613 in rabbits: long-term study

Arthritis was induced in the 24 rabbits as described above. Two days after the induction of arthritis in the rabbits [20–22], 5 mg/joint SI-613 or PBS was administered at a volume of 500 μL/joint into the knee joint cavity. Concentrations of DF in the synovium and synovial lavage fluid were measured by LC-MS/MS on days 1, 3, 7, 14, 21, 28, 35, and 42 after a single IA administration.

#### Statistical analyses

Statistical analyses were performed using the Statistical Analysis System, SAS (SAS Institute Inc., Cary, NC). Direct effects from treatment with SI-613 were assessed using two-way analyses of variance followed by Williams’ or Tukey’s test for the analgesic assessment of the joint pain model in rats. A Student’s *t*-test, Welch’s *t*-test, or Tukey’s test was performed for the assessment of the PGE_2_ content in the SF. A Student’s *t*-test with Holm’s correction was used for the assessment of long-lasting anti-inflammatory effects. Results of the measurements in each group were represented as the mean and 95% confidence intervals (CI) for the pharmacological study or standard deviations (SD) for the pharmacokinetic study; *p* values of < 0.05 were considered statistically significant.

## Results

### Analgesic effects of SI-613 on silver nitrate-induced arthritic pain in rats

**Fig. 2:**
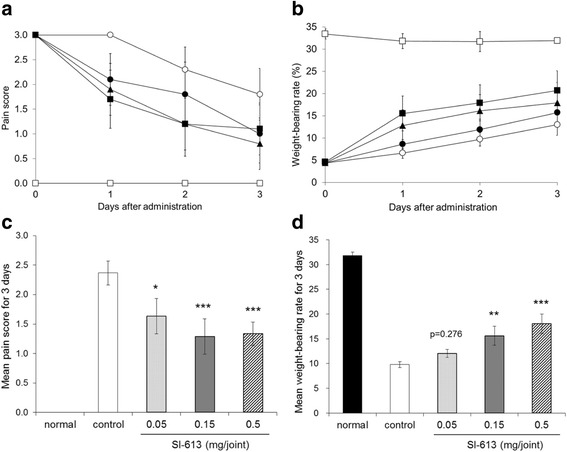
Analgesic effect of SI-613 in silver nitrate-induced arthritic pain model in rats. Silver nitrate-induced arthritic rats given 0.05 mg (closed circle), 0.15 mg (closed triangle), 0.5 mg (closed square) SI-613, or vehicle (open circle) intra-articularly, and non-treated normal rats (open square) were evaluated for pain score (**a**) and weight-bearing rate (**b**) over time. Mean values of pain scores (**c**) and weight-bearing rate (**d**) for 3 days were calculated and subjected to statistical analysis: two-way analyses of variance followed by Williams’ test. ***p < 0.005, **p < 0.01, *p < 0.05 (vs. control, significant level at 5%, two-tailed). Values represent the means ±95% confidence intervals (*n* = 9 per group, except for the normal group *n* = 3)

**Fig. 3:**
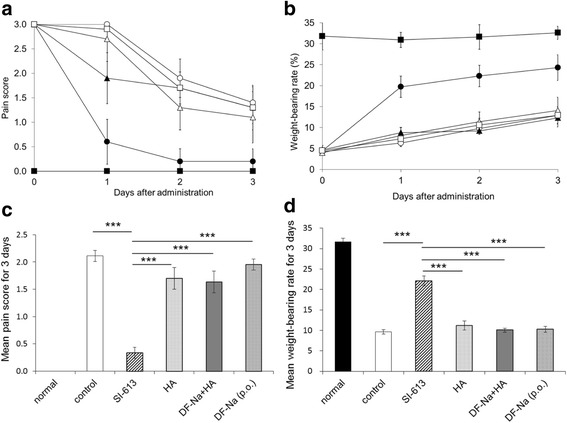
Analgesic effects of SI-613 and its chemical compositions in silver nitrate-induced arthritic pain model in rats. Silver nitrate-induced arthritic rats given SI-613 (closed circle), HA (open triangle), DF-Na + HA (closed triangle), or vehicle (open circle) intra-articularly; those given DF-Na orally once daily for 3 days (open square); and non-treated normal rats (closed square) were evaluated for pain score (**a**) and weight-bearing rate (**b**) over time. Mean values of pain scores (**c**) and weight-bearing rate (**d**) for 3 days were calculated and subjected to statistical analysis: two-way analyses of variance followed by Tukey’s test. ***p < 0.001 (vs. SI-613, significant level at 5%, two-tailed). Values represent the means ±95% confidence intervals (*n* = 9 per group, except for the normal group *n* = 3)

SI-613 improved the pain behavioral scores significantly at doses of 0.05, 0.15, and 0.5 mg/joint in a dose-dependent manner (Fig. 2a and c). In addition, SI-613 at 0.15 and 0.5, but not at 0.05 mg/joint significantly increased the weight-bearing rates in a dose-dependent manner compared with those in the control group (Fig. 2b and d). Furthermore, compared with PBS, HA, or DF-Na + HA, or the repeated oral administration of DF-Na, SI-613 significantly improved the pain behavioral score (Fig. 3a and c) and increased the weight-bearing rate (Fig. 3b and d). On day 1, the pain score in the DF-Na + HA group was lower than that in the control group; however, this analgesic effect was not observed on and after day 2. It is concluded that a single IA administration of SI-613 exerts a more efficacious and longer-lasting analgesic effect for arthritic pain than that exerted by individual chemical compositions.

**Fig. 4:**
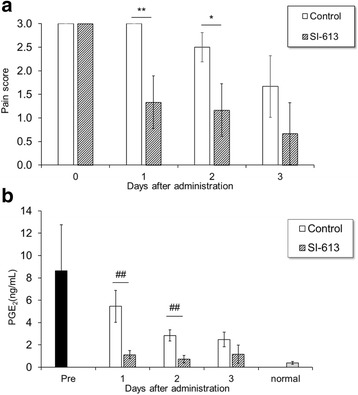
Effects of SI-613 on PGE_2_ content in the synovial fluid of silver nitrate-induced arthritic rats. Silver nitrate-induced arthritic rats given 0.5 mg SI-613 or vehicle intra-articularly were evaluated for pain score (**a**), and sacrificed for measurement of the prostaglandin E_2_ (PGE_2_) content in the SF (**b**) on days 1, 2, and 3. For statistical analysis of pain score, Wilcoxon test was used. **p < 0.01, *p < 0.05 (vs. control, significant level at 5%, two-tailed). For statistical analysis of the PGE_2_ content, Welch’s *t*-test was used. ##*p* < 0.01 (vs. control, significant level at 5%, two-tailed). Values represent the means ±95% confidence intervals [*n* = 6 per group, except for the normal group (PGE_2_ content) *n* = 7]

In addition, the anti-inflammatory effect of SI-613 was assessed by measuring the PGE_2_ content in the SF. The PGE_2_ content decreased over time, but high values were maintained for 3 days in this animal model. SI-613 group showed lower PGE_2_ content than the control group (Fig. 4b). The analgesic effect was confirmed each day (Fig. 4a).

### Effect of SI-613 on the PGE_2_ content in the SF of rabbits with antigen-induced arthritis

**Fig. 5:**
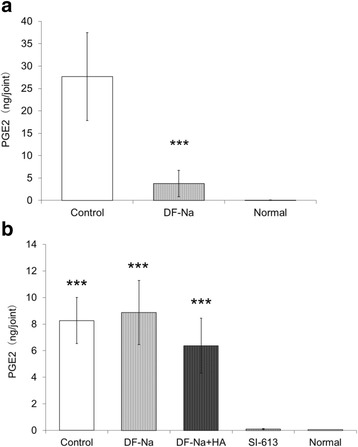
Effects of SI-613 on PGE_2_ content in the synovial fluid of antigen-induced arthritis in rabbits. Two days after the induction of arthritis, the test materials were administered. The synovial fluid (SF) was collected at 3 (**a**) or 72 (**b**) hours after the administration of the test materials. The prostaglandin E_2_ (PGE_2_) content in the SF was measured with a PGE_2_ enzyme-linked immunosorbent assay (ELISA) kit. For statistical analysis, a Student’s *t*-test and Tukey’s test were used for the 3 h and 72 h data, respectively. **a** ***p < 0.001 (vs. control, significant level at 5%, two-tailed). Values represent the means ±95% confidence intervals. (*n* = 10 per group, except for the normal group *n* = 5) (**b**) ***p < 0.001 (vs. SI-613, significant level at 5%, two-tailed). Values represent the means ±95% confidence intervals. (*n* = 10 per group, except for the normal group *n* = 5)

At 3 h after administration, the mean PGE_2_ content in the SF was 27,651 pg/joint (95% CI = 17,844–37,458 pg/joint) in the control group, whereas that in the DF-Na group was 3767 pg/joint (95% CI = 847–6687 pg/joint) (Fig. 5a). Orally administered DF-Na significantly suppressed the production of PGE_2_ in the SF compared with the control group, suggesting that this model was appropriate for measuring PGE_2_. At 72 h after the injection, the mean PGE_2_ content in the SF was 8267 pg/joint (95% CI = 6535–9999 pg/joint) in the control group. In the DF-Na, DF-Na + HA, and SI-613 groups, the PGE_2_ content was 8873 pg/joint (95% CI = 6464–11,282 pg/joint), 6378 pg/joint (95% CI = 4319–8437 pg/joint), and 106 pg/joint (95% CI = 70–142 pg/joint), respectively. SI-613 significantly suppressed the production of PGE_2_ in the SF compared with that of DF-Na, DF-Na + HA, and the control group (Fig. 5b). No significant differences were observed between the DF-Na, DF-Na + HA, and control groups. A single IA administration of SI-613 was shown to exert a longer-lasting effect compared with DF-Na or DF-Na + HA.

### Long-lasting anti-inflammatory effect of SI-613 on antigen-induced arthritis in rabbits

**Fig. 6:**
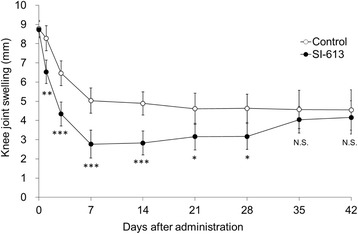
Long-lasting anti-inflammatory effect of SI-613 on the knee joint swelling of antigen-induced arthritic rabbits. Two days after the induction of arthritis, 1% SI-613 or PBS was administered at a volume of 500 μL/joint into the joint cavities. The knee joint swelling was evaluated on the day before (day 0), and on days 1, 3, 7, 14, 21, 28, 35, and 42 after the injection of test materials. For statistical analysis, a Student’s *t*-test with Holm’s correction was used. ***p < 0.001, **p < 0.01, *p < 0.05 (vs. control, significant level at 5%, two-tailed). N.S., not significant. Values represent the means ±95% confidence intervals. (*n* = 20 per group)

SI-613 significantly decreased the knee joint swelling compared with the control on day 1 after the injection, and exerted an anti-inflammatory effect continuously until day 28 (Fig. 6). However, on day 35 and day 42, there was no significant difference in the joint swelling of SI-613-treated and control animals.

### Distribution of DF in knee tissues after a single administration of SI-613 and its chemical compositions in rabbits: short-term study

In the antigen-induced arthritis model, the DF concentrations in the synovium and synovial lavage fluid were determined at 72 h after a single IA administration of SI-613 or a mixture of DF-Na and HA. In addition, the DF concentrations at 3 and 72 h after the oral administration of DF-Na were determined. As shown in Table 1, the SI-613-treated group showed higher DF concentrations in the synovium and synovial lavage fluid than the other groups at 72 h after the injection.

**Table 1:** DF concentrations in the synovium and synovial joint cavity of rabbits with antigen-induced arthritis

Group	Sampling point	Animal Number	Concentration of DF	
			Synovium (ng/g tissue)	Synovial lavage fluid (ng/mL fluid)
DF-Na (oral)	3 h	1	155.8	124.6
		2	52.88	58.23
		3	75.83	45.66
	72 h	4	BLQ1	BLQ2
		5	BLQ1	BLQ2
		6	BLQ1	BLQ2
A mixture of DF-Na and HA (IA)	72 h	1	BLQ1	BLQ2
		2	BLQ1	BLQ2
		3	48.02	21.70
SI-613 (IA)	72 h	1	15.23	60.64
		2	45.47	38.82
		3	351.1	1540

BLQ1: below limit of quantification, < 5 ng/g

BLQ2: < 1 ng of DF per mL of synovial lavage fluid

Time for calculation of area under the DF plasma concentration-time curve (AUC_0-t_), t (day), was the latest time point at which DF was quantifiable. The half-life (T_1/2_) was determined by semi-log plotting the data of at least three time points after T_max_. T_1/2_, AUC_0-∞_, and AUC_0-t_ were not obtained for one animal in the DF-Na + HA group and all animals in the SI-613 group, both of which did not provide a required number of effective time points after T_max_, and shown as NC (not calculated). The maximum plasma concentration (C_max_) of DF in the SI-613 group (IA) was 462 and 94 times lower than those of the DF-Na group (oral) and DF-Na + HA group (IA), respectively. Similarly, the AUC_0-t_ of the SI-613 group (IA) was 187 and 16 times smaller than that of the DF-Na group (oral) and DF-Na + HA group (IA), respectively (Table 2).

**Table 2:** Plasma DF concentrations of rabbits with antigen-induced arthritis

Group	C_max_ (ng/mL)	T_max_ (h)	AUC_0-t_ (ng·h/mL)	AUC_0-∞_ (ng·h/mL)	t_1/2_ (h)
DF-Na (oral)	621.0 ± 371.6	2.4 ± 3.2	4693 ± 1905	4132^b^	12.8^b^
Mixture^a^	125.9 ± 68.5	0.39 ± 0.53	393.4 ± 167.6	423.2 ± 154.5	3.4 ± 2.0
SI-613 (IA)	1.343 ± 0.050	24 ± 0	25.11^b^	NC	NC

Mean ± SD (*n* = 3 or *n* = 2) calculated from the individual PK parameters

*NC* Not calculated.

BLQ: < 1 ng/mL

^a^ a mixture of DF-Na and HA (IA)

^b^ *n* = 2

### Evaluation of DF in knee tissues after a single IA administration of SI-613 in rabbits: long-term study

**Fig. 7:**
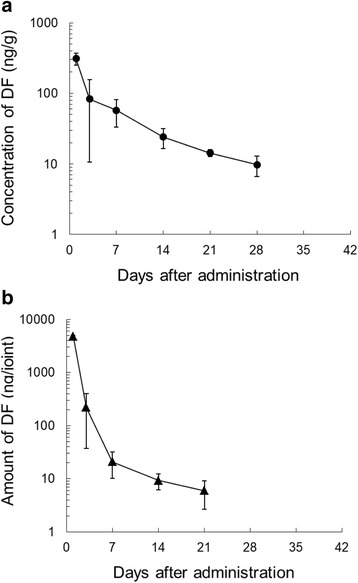
**a** Profile of diclofenac sodium (DF) concentrations in the synovium after a single IA administration of SI-613 in rabbits with antigen-induced arthritis. SI-613 was intra-articularly injected at a dose of 5 mg/joint, and each value shows the mean ± standard deviation (S.D.) of 3 animals. The lower limit of quantification was 5 ng/g. **b** Profile of DF concentrations in the joint cavity after a single IA administration of SI-613 in rabbits with antigen-induced arthritis. SI-613 was intra-articularly injected at a dose of 5 mg/joint, and each value shows the mean ± S.D. of 3 animals. The lower limit of quantification was 1 ng of DF per mL of synovial lavage fluid

Concentrations of DF in the synovium and synovial lavage fluid of antigen-induced arthritis rabbits were determined after a single IA injection of 5 mg of SI-613 by LC-MS/MS. The mean concentration in the synovium was 9.754 ng/g on day 28 and decreased below the lower limit of quantification (< 5 ng/g) on day 35 (Fig. 7a). The mean amount of DF in the joint cavity lavage fluid was 5.940 ng/joint on day 21 and decreased below the lower limit of quantification (< 1 ng of DF per mL of synovial lavage fluid) on day 28 (Fig. 7b). The pharmacokinetic parameters of DF are listed in Table 3.

**Table 3:** PK parameters of DF after single IA administration of SI-613 in rabbits with antigen-induced arthritis

	C_max_ (ng/g)	T_max_ (day)	AUC_0-28day_ (ng∙day/g)	AUC_0-∞_ (ng∙day/g)	t_1/2_ (day)
Synovium	311.6	1	1336	1487	10.8^a^
	C_max_ (ng/joint)	T_max_ (day)	AUC_0-21day_ (ng∙day/joint)	AUC_0-∞_ (ng∙day/joint)	t_1/2_ (day)
Synovial lavage fluid	4726	1	7956	8018	7.7^b^

SI-613 was intra-articularly injected at a dose of 5 mg/joint. The PK parameters were calculated from mean concentrations of DF in synovium or mean amounts of DF in joint cavity (*n* = 3)

^a^, Calculated from 14 to 28 days after administration

^b^, Calculated from 7 to 21 days after administration

## Discussion

We have investigated the novel HA derivative chemically linked with DF, SI-613, which is a potentially safer and more effective treatment for OA knee pain. In the present study, the pharmacological effects of SI-613 were comprehensively evaluated by comparing the pain response, weight-bearing rate on the inflamed legs, joint swelling, and PGE_2_ content in the SF as an indicator of hydrarthrosis using rat and rabbit arthritis models. In the arthritic pain model, it was shown that a single IA injection of SI-613 exerted an analgesic effect more effectively than orally administered DF-Na in a dose-dependent manner. The analgesic effect of SI-613 was most probably due to inhibition of PGE_2_ production, although the effect on it was slightly varying depending on the model. The PGE_2_-inhibitory effect was statistically significant at 72 h after SI-613 administration in the rabbit model but not in the rat model. This can be attributed to the difference of the dynamic range between the models. In the rabbit models, the difference of normal and control groups was sufficiently large even at 72 h. On the other hand, the PGE_2_ level of the control group in the rat model was only about 6 times larger than that of the normal group, and then there was no statistically significant difference between the SI-613 and control groups. Moreover, the effect of SI-613 was long-lasting, which was never achieved with the DF-Na + HA mixture. The unstable analgesic effect of uncombined-DF-Na (without HA) is probably due to the fact that it is not well retained in the synovium, which is a therapeutic target tissue for OA. In contrast, it is most likely that intra-articularly administered SI-613 is retained in the synovium for a prolonged period and sustainably releases DF in the inflamed region. The relevance of this notion is supported by other studies that found that HA administered intra-articularly to the joint cavity penetrated into the synovium and remained in the synovium for a longer period [23, 24]. Furthermore, this efficient retention in the synovium might benefits from the high affinity of HA and its cell surface receptor, CD44 [25], expressed in the synovium [26]. SI-613, thus, delivered efficacious concentrations of DF to synoviocytes. Therefore, we consider that HA is an indispensable component for the analgesic effect of SI-613.

The superiority of SI-613 was supported by its inhibitory effect on the production of PGE_2_. This effect resulted from DF being sustainably released from SI-613. The relationship between the pharmacological effects and pharmacokinetics of SI-613 was investigated using a rabbit antigen-induced arthritis model. SI-613 showed a sustainable anti-inflammatory effect for 72 h after administration, whereas the mixture of DF and HA or oral DF-Na did not. The concentration of DF in the synovium and SF at 72 h after the administration of SI-613 was higher than that after the administration of the mixture of DF and HA or oral DF-Na. SI-613 exerted a long-lasting analgesic effect, for 28 days. However, no significant difference was observed on day 35 and 42. The DF concentration in the synovium reached its maximum level of 311.6 ng/g on day 1 after the injection and this gradually declined to 10 ng/g by day 28. It fell below the lower limit of quantification (< 5 ng/g) on day 35. Therefore, there is a clear correlation between the analgesic effect and the retention period of DF. The DF concentrations at 28 days after the SI-613 administration were at comparable levels with those in humans after repeated administration of DF preparations. In the clinical studies of current DF preparations, the DF concentrations in the synovium ranged from 5 to 35 ng/g after repeated oral administration of DF tablets and hard capsules or repeated topical administration of DF gel ointments and cataplasms [27, 28]. These findings suggest that SI-613 exerted an analgesic effect via the sustained release of DF, and this pharmacological effect lasted for at least 28 days.

It has been reported that HA inhibits the phosphorylation of p38 mitogen-activated protein kinase (MAPK) via its principal receptor CD44 and exerts an anti-inflammatory effect [29]. Furthermore, HA inhibits the production of PGE_2_, and pretreatment with OS/37, a monoclonal antibody specific for the hyaluronate-binding epitope on CD44, reversed the inhibitory effects of HA [30]. The inhibition of the production of PGE_2_ by HA was also confirmed in a clinical study [31]. In addition, it was reported that HA exerted an analgesic effect by covering free nerve endings in articular tissues such as the synovial membranes, menisci, and ligaments [32]. This suggests that SI-613 exerts a clearly superior analgesic effect than DF or HA, or co-administered DF + HA.

Oral NSAIDs are widely prescribed for the relief of OA pain, however, upper gastrointestinal tract complications have been reported in patients who received long-term oral NSAIDs [2]. NSAIDs are also considered to have limited efficacy for OA pain relief. In 1990s, a number of selective COX-2 inhibitors were developed to reduce the adverse events of NSAIDs. However, most of them were withdrawn from the market after the cardiovascular adverse effects of COX-2 inhibitor were reported in 2004 [3]. Meanwhile, oral NSAIDs including DF-Na were also reported to have the same concerns as COX-2 inhibitors [4, 5]. In the present study, the C_max_ of DF (1.343 ng/mL) in the animals given a single IA effective dose of SI-613 was 462 times lower than that in the animals given a single oral effective dose of DF-Na. The AUC_0-t_ (25.11 ng·h/mL) of the SI-613-treated group was 187 times lower than that of the oral DF-Na-treated group. Moreover, the DF concentrations after the SI-613 injection were lower than the reported values of DF-Na after oral administration to humans at the clinical dose (3 × 50 mg/day) [33]. Additionally, the pharmacokinetics of Voltaren^®^ Gel (1% diclofenac sodium topical gel) has been assessed in healthy volunteers following repeated applications to 1 knee [4 × 4 g per day (= 160 mg DF-Na per day)] for 7 days [33]. The C_max_ of DF was 15 ± 7.3 ng/mL, and the value is comparable with those of SI-613-treated animals. These results indicate that systemic toxicities are unlikely to be attributable to DF after a single IA administration of SI-613 to the knees.

OA is characterized by gradual cartilage degeneration. Although NSAIDs are effective in relieving OA pain and have been in use for decades, it remains controversial as to what effects NSAIDs have on the progression of OA. Reijman et al. made the observation that the chronic use of DF, but not ibuprofen, naproxen, or piroxicam, accelerated the progression of knee and hip OA in subjects aged over 55 years [34]. In another paper, Huskisson et al. reported that indomethacin increases the rate of radiological deterioration in the knee joint space of patients with OA [35]. However, the beneficial or neutral effects of NSAIDs have been reported in in vitro and in vivo studies [36–39]. de Boer et al. reported that celecoxib, a selective COX-2 inhibitor, has a chondroprotective effect [40]. Therefore, it is still unclear whether NSAIDs cause cartilage degeneration. IA-HA can protect against articular cartilage degeneration of the knee accelerated by NSAIDs (loxoprofen monosodium and indomethacin) via the inhibition of matrix metalloproteinase (MMP) production [41, 42]. MMPs are upregulated in the chondrocytes of human OA [43–45] and play a critical role in cartilage destruction by degrading collagen and aggrecan, the main proteoglycan of chondrocyte. SI-613, containing HA as a component, may inhibit the interleukin-1β-stimulated production of MMP-1, − 3 and − 13 in human chondrocytes as well as HA. Future research agendas on SI-613 should involve clarifying the pharmacological effect of HA moiety for SI-613.

## Conclusion

In conclusion, our results show that a single IA administration of SI-613 provides an efficacious and safe treatment for OA pain with a potent and long-lasting analgesic effect compared to existing IA-HA injections or oral NSAIDs.

## Abbreviations

CI: Confidence intervals
COX-2: Cyclooxygenase-2
DF: Diclofenac
FCA: Freund’s complete adjuvant
HA: Hyaluronic acid
IA: Intra-articular
LC-MS/MS: High performance liquid chromatography coupled with tandem mass spectrometry
MAPK: Mitogen-activated protein kinase
MMP: Matrix metalloproteinase
NSAIDs: Nonsteroidal anti-inflammatory drugs
OA: Osteoarthritis
OVA: Ovalbumin
PBS: Phosphate-buffered saline
PGE_2_: Prostaglandin E_2_
SD: Standard deviations
SF: Synovial fluid
